# Respectful maternal and newborn care: measurement in one EN-BIRTH study hospital in Nepal

**DOI:** 10.1186/s12884-020-03516-4

**Published:** 2021-03-26

**Authors:** Rejina Gurung, Harriet Ruysen, Avinash K. Sunny, Louise T. Day, Loveday Penn-Kekana, Mats Målqvist, Binda Ghimire, Dela Singh, Omkar Basnet, Srijana Sharma, Theresa Shaver, Allisyn C. Moran, Joy E. Lawn, Ashish KC, EN-BIRTH Study Group

**Affiliations:** 1 Research Division, Golden Community, Lalitpur, Nepal; 2 Centre for Maternal, Adolescent, Reproductive & Child Health (MARCH), London School of Hygiene & Tropical Medicine, London, UK; 3 Department of Women’s and Children’s Health, Uppsala University, Dag Hammarskjölds väg 14B, Uppsala, Sweden; 4 Ministry of Health and Population, Kathmandu, Nepal; 5 USAID (contractor), Washington, DC, USA; 6 Department of Maternal, Newborn, Child and Adolescent Health and Ageing, WHO, Geneva, Switzerland

**Keywords:** Respectful maternal and newborn care, Mistreatment, Nepal, Maternal, Newborn, Coverage, Respect, Privacy, Delivery, Standard of care

## Abstract

**Background:**

Respectful maternal and newborn care (RMNC) is an important component of high-quality care but progress is impeded by critical measurement gaps for women and newborns. The *Every Newborn* Birth Indicators Research Tracking in Hospitals (EN-BIRTH) study was an observational study with mixed methods assessing measurement validity for coverage and quality of maternal and newborn indicators. This paper reports results regarding the measurement of respectful care for women and newborns.

**Methods:**

At one EN-BIRTH study site in Pokhara, Nepal, we included additional questions during exit-survey interviews with women about their experiences (July 2017–July 2018). The questionnaire was based on seven mistreatment typologies: Physical; Sexual; or Verbal abuse; Stigma/discrimination; Failure to meet professional standards of care; Poor rapport between women and providers; and Health care denied due to inability to pay. We calculated associations between these typologies and potential determinants of health – ethnicity, age, sex, mode of birth – as possible predictors for reporting poor care.

**Results:**

Among 4296 women interviewed, none reported physical, sexual, or verbal abuse. 15.7% of women were dissatisfied with privacy, and 13.0% of women reported their birth experience did not meet their religious and cultural needs. In descriptive analysis, adjusted odds ratios and multivariate analysis showed primiparous women were less likely to report respectful care (β = 0.23, *p*-value < 0.0001). Women from Madeshi (a disadvantaged ethnic group) were more likely to report poor care (β = − 0.34; *p*-value 0.037) than women identifying as Chettri/Brahmin. Women who had caesarean section were less likely to report poor care during childbirth (β = − 0.42; *p*-value < 0.0001) than women with a vaginal birth. However, babies born by caesarean had a 98% decrease in the odds (aOR = 0.02, 95% CI, 0.01–0.05) of receiving skin-to-skin contact than those with vaginal births.

**Conclusions:**

Measurement of respectful care at exit interview after hospital birth is challenging, and women generally reported 100% respectful care for themselves and their baby. Specific questions, with stratification by mode of birth, women’s age and ethnicity, are important to identify those mistreated during care and to prioritise action. More research is needed to develop evidence-based measures to track experience of care, including zero separation for the mother-newborn pair, and to improve monitoring.

**Supplementary Information:**

The online version contains supplementary material available at 10.1186/s12884-020-03516-4.

## Key findings

**Table:** 

**What is known and what is new about this study?** • Whilst ~ 80% of births globally are now in health facilities; previous studies have estimated that 19–98% women worldwide experience disrespect and abuse during facility birth. • The experience of care for women, newborns and their families around the time of birth is increasingly recognised as a global priority and an essential dimension of quality of care, but accurate measurement, and especially routine tracking are challenging. • This study in Pokhara Nepal (an EN-BIRTH study site), captured respectful maternal and newborn care (RMNC) in exit-survey after hospital birth using seven typologies (*n* = 4296). **Measurement—what did we find and what does it mean?** • *Standards of care showed a wide range:* we found very low exit-survey reported coverage of specific questions regarding standards of care, such as 0.3% of women having a companion of choice and 0.5% having skin-to-skin contact with their baby. This contrasted with consistently high (100%) women’s exit-survey report regarding treatment with dignity and respect, or absence of abuse. • *Question design mattered:* When asked more general survey questions, all women denied physical/sexual/verbal abuse, and expressed they had been treated with respect and dignity. However, more specific questions including regarding preservation of privacy, support meeting religious/cultural needs, access to their chosen birth companion, skin-to-skin contact, and breastfeeding counselling after birth revealed gaps in service provision. • *Women’s characteristics:* Primiparous women were more likely to report non-respectful care. • *Variation with mode of birth:* Women who had caesarean had a 98% decrease in the odds (aOR = 0.02, 95% CI, 0.01–0.05) of receiving skin-to-skin contact with their baby than those with vaginal births. Women who had caesarean were more likely to report respectful care during childbirth (β = − 0.402; *p*-value < 0.0001) than women with vaginal births. **What next and research gaps?** • Exit interview surveys underestimate a negative experience of care, even with an independent interviewer. Further improvements in measurement of more tangible events (privacy, companionship, separation) in large-scale household surveys linked to other data sources (such as service readiness surveys) are needed. • Specific indicator measurement testing including validity for experience of newborn care (e.g. skin-to-skin contact as a proxy for zero separation) could be assessed for potential use as a tracer indicator of RMNC in different information systems. • Considering the profile of the family and the mode of birth are important to capture inequalities in respectful care and to prioritise gaps for action. • Research is needed to understand if improving experience of care for vaginal births may help curb rising caesarean section rates.	

## Background

Annually, almost 80 million babies are now born in health facilities [1], a 50% increase in the last 20 years especially in low- and middle-income countries (LMICs) [2]. This is a major result of key investments to bring global attention to improving women’s health [3], with an additional 3 million maternal and neonatal deaths estimated to have been averted in 2018 [4]. However, poor quality of care at the time of facility birth remains a contributor to around 66% of the 2.4 million neonatal deaths globally each year [4–6]. High-quality health systems with quality of care for facility birth could prevent an estimated 1 million newborn deaths and half of all maternal deaths every year [7].

Quality of care has two dimensions – provision and experience of care [8, 9]. Provision of quality care is essential and describes the content and quality of clinical interventions and services. However, without a positive user experience across all domains of the WHO respectful care framework [9], families may lose trust in services. Evidence shows that women who were mistreated during labour and birth are hesitant to engage with postnatal services, irrespective of whether provision of care is in accordance with clinical guidelines [8, 10, 11]. Many studies in the last decade have highlighted mistreatment of women during labour in LMICs [12–14], including physical and verbal abuse, discrimination based on maternal age (young or elderly), and ethnicity or social class [15, 16]. Other manifestations of mistreatment included the provision of care without consent, obstructing the presence of a birth companion, and withholding food during labour without the woman’s consent or a clinical indication [15, 17]. In contrast, respectful care is synonymous with a positive user experience and should include women and families as active-participants throughout pregnancy and childbirth [18]. Respectful care for newborns is a more recent concept; efforts are being made to define and agree on an expanded typology of respectful care that is more inclusive of the newborn [19]. The White Ribbon Alliance’s (WRA) Respectful Maternity Care Charter outlines the rights of women and newborns during childbirth and the postnatal period [20], but there is very limited evidence regarding how to measure such inclusive respectful maternal and newborn care (RMNC) in practice.

WRA outlines that provision of respectful care demands health systems, services and workers are able to meet families’ cultural and religious needs [20]. These are often defined by local culture, traditions and beliefs that influence the choice of birth place, preference of support person, and a woman’s sense of control and safety [21]. In Nepal, as in many settings, cultural beliefs and practices around childbirth vary between different communities and create both opportunities and barriers for uptake of services and interventions (e.g. facility birth) [22]. This adds complexity when considering implementation approaches and envisioning contextually relevant, validated measurement tools to track RMNC.

Although emergency caesarean section can be a life-saving intervention for a woman or her baby facing complications during labour, escalating global caesarean rates suggest overuse in both high- and low-resource settings [23–26]. In recent years, the southeast Asian region has seen the caesarean rate increase from 4.4 to 19.2% [25], with Nepal highlighted as one of the countries with the highest increase in caesarean rates, especially among the richest fifth of the population [23]. However, little is yet known about how mode of birth impacts the family experience of care, or the measurement of RMNC.

Improving RMNC requires a health systems approach to support frontline health workers’ capacity to facilitate a positive experience of care [27]. A recent study highlighted that many health systems struggle to support family/woman-centred care [17]. This gap in service provision could risk a decline in facility births, and reverse the hard-won momentum for improving outcomes for maternal and newborn survival and reducing stillbirths. Despite this, a recent review of facility assessment tools found that measures of care experience were least likely to be included [28].

Tracking progress on respectful care is necessary to improve quality of care, but currently there is a lack of consensus regarding what is best to measure based on the WHO standards of care and specific goals and targets [9, 29]. Moreover, there is limited evidence on the different measurement options, including exit interviews after facility births, household surveys, independent observation, or capturing respectful care in routine health management information systems (HMIS) [12]. Concerns exist that implementation of poor data collection methods to capture these complex and sensitive data [30] result in an underestimate of the true prevalence [31].

The *Every Newborn* Action Plan (ENAP) agreed by all United Nations member states and > 80 development partners, includes an ambitious measurement improvement roadmap with an urgent focus on validating measurement of indicators for care and outcomes around the time of birth [32]. As part of this roadmap, The *Every Newborn* - Birth Indicators Research in Hospitals (EN-BIRTH) study was a mixed-methods observational study of > 23,000 facility births in three countries (Tanzania, Bangladesh and Nepal). EN-BIRTH aimed to test the validity of measurement for selected newborn and maternal indicators for routine facility-based tracking of coverage and quality of care [32]. Data were collected in collaboration with the Nepal Perinatal Quality Improvement Project (NePeriQIP) [33, 34].

### Objectives

This paper is part of a supplement based on the EN-BIRTH multi-country validation study, ‘*Informing measurement of coverage and quality of maternal and newborn care*’. We focus on exit survey-reported RMNC at one EN-BIRTH study site in Nepal, with three objectives:

1. **Analyse EXIT SURVEY-REPORTED EXPERIENCE OF CARE FOR WOMEN** after hospital birth (selected maternal respectful care components, based on Bohren et al. [12]).
2. **Describe women’s EXIT SURVEY-REPORTED COVERAGE OF FACILITY-BASED NEWBORN CARE** practices around the time of birth (selected newborn respectful care components).
3. **Conduct multivariate regression analysis regarding DETERMINANTS OF SURVEY-REPORT** by women, including mode of birth, and demographic and social characteristics.

## Methods

EN-BIRTH was an observational mixed-methods study to validate measurement of selected maternal and newborn indicators in survey and routine recording. Data were collected between July 2017 and July 2018 in five public hospitals providing comprehensive emergency obstetric and neonatal care (CEmONC) in three high-burden countries: Bangladesh, Nepal and Tanzania. Detailed information regarding the research protocol, methods and analysis has been published separately [32]. This paper focuses on the measurement of respectful care of women and newborns, obtained from exit surveys, at Pokhara Academy of Health Sciences, where questions pertaining to RMNC were added to the standard EN-BIRTH exit interview survey as part of the NePeriQIP project [33].

Women were recruited in early labour and voluntary informed written consent was obtained from all study participants. Participants were assured of anonymity and confidentiality, although there were recognised challenges for using facility-based survey tools for this topic. Results are reported in accordance with the STROBE Statement checklist for cross-sectional studies (Additional file 1).

### Tool development and data collection

For this study, women’s experience of care during childbirth and sociodemographic information were collected using a semi-structured questionnaire administered at the time of discharge. We used 11 questions to assess mistreatment of women and newborns during childbirth and the postnatal period using the “abuse and disrespect” typology based on a systematic review by Bohren et al. [12] (Additional file 2). The respectful maternity care structured questionnaire was designed in English, translated into Nepali, then independently back-translated and finalised after pilot testing [35]. Data were collected on paper-based forms and checked for completeness. Every month, researchers observed a 5% sample of data collector interviews in order to assess adherence to the research protocol. Feedback and training were provided to data collectors when necessary. Data were digitalized and stored in the CS-PRO database. Data were backed up weekly using an external hard drive and stored in a locked vault. Paper forms were stored in locked cabinets as per the data security protocol. Women who consented to be part of this study were tracked from admission until discharge. Community follow-up was not possible and is a noted limitation of this study. All caesarean sections were undertaken using epidural anaesthesia.

### Objective 1: Respectful maternal care

A descriptive analysis on the coverage gaps for respectful maternity care was done based on the seven typologies of mistreatment for which we could collect data [12]:

(1) Physical, (2) Sexual, and/or (3) Verbal abuse

- Were you or your newborn physically, verbally or sexually abused during labour or childbirth or after birth?
- Were you treated in a bad way?

(4) Stigma and discrimination

- Did the health service meet your religious and cultural birthing practice needs?
- Were you treated with respect?
- Was your dignity preserved during your stay at the hospital?

(6) Poor rapport between woman and provider

- Ineffective communication
  – Were you satisfied with the health education and information you received from health care providers?
  – Were you given the opportunity to discuss any concerns and preferences?
- Were you satisfied with the degree of privacy received during your stay at the hospital?

(7) Health system constraints

- Were you refused care because of your inability to pay?
- Were you satisfied with the degree of privacy received during your stay at the hospital?

### Objective 2: Respectful newborn care

A descriptive analysis on the coverage gaps for respectful newborn care was done based on the seven typologies of mistreatment for which we could collect data [12]:

(1) Physical, (2) Sexual, and/or (3) Verbal abuse

- Were you or your newborn physically, verbally or sexually abused during labour or childbirth or after birth?
- Were you treated in a bad way?

(5) Failure to meet professional standards of newborn care

- Did you keep your baby in skin-to-skin contact immediately after birth?
- Did a health worker examine your baby when you were present?

(6) Poor rapport between woman and provider

- Ineffective communication
  – Did you receive written or verbal information and counselling on exclusive breastfeeding until 6 months before discharge?

### Objective 3: Association between reporting of poor care with socio-demographic and obstetric characteristics

Amongst mothers who reported mistreatment of themselves or their newborn, a test of association with age, ethnicity and mode of birth was done using an unpaired student t-test. Categorical variable groups were made for age, ethnicity, parity and mode of birth. Two groups were identified based on ethnicity/religion: an advantaged group (women identifying as Chettri/Brahmin and others) and a disadvantaged group (participants identifying as Dalit; Janjati; Madhesi or Muslim) [34, 36]. Parity data were combined into three groups (no previous birth, 1 previous birth, and 2+ previous births). Mode of birth was analysed by vaginal birth (spontaneous or assisted) and caesarean section births. Missing values in each variable were reported and excluded from this analysis. We have excluded data with very high (> 90%) or low (< 10%) proportions of “Yes” replies resulting in low variance (< 10%).

Multivariable logistic regression models were fitted to evaluate whether age, ethnicity, mode of birth, parity or baby’s sex could be a predictor of women reporting non-respectful care. If any level of association was observed in the logistic regression analysis, the variables were taken for multi-nominal regression analysis, which included women’s reports of whether the health service met religious and cultural birthing practice needs, and privacy during the hospital stay. We prioritised results from the multivariate logistic regression model above the adjusted odds ratios.

## Results

**Fig. 1:**
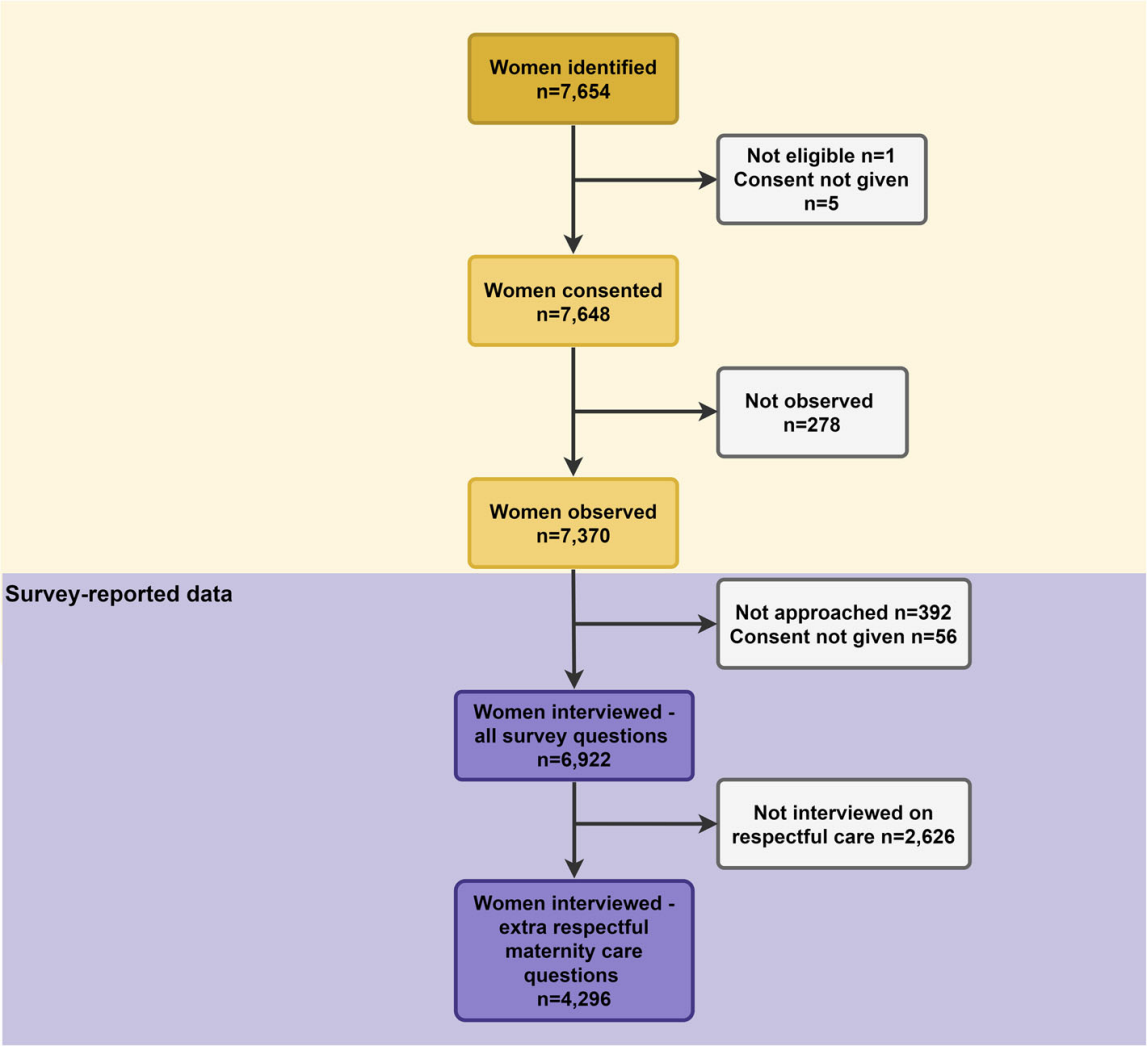
Flow diagram for respectful maternal and newborn care in Pokhara Hospital, EN-BIRTH study (*n* = 7370)

During the study period, 6922 women had exit interviews for the NePeriQIP study, of which 4296 (62.1%) ID-matched for the EN-BIRTH study and are reported here (Fig. 1). The mean age of exit-survey respondents was 24 years, 48.1% of participants identified as Chettri/Brahmin, and > 90% of women gave birth at term (Table 1, Additional file 3). We report results in accordance with the disrespect and abuse typologies (Table 2).

**Table 1:** Background characteristics of women, EN-BIRTH study (*n* = 4296)

		EN-BIRTH
	N	Proportion (95% CI)
**Age (mean ± SD)**	4296	24.3 ± 4.5
**Woman’s age**		
< 20 yrs	563	13.1 (12.1, 14.1)
20–29 yrs	3149	73.3 (72.0, 74.7)
≥ 30 yrs	584	13.6 (12.6, 14.6)
**Parity**		
No previous birth	619	14.4 (13.4, 15.5)
One previous birth	1924	44.8 (43.3, 46.3)
Two or more previous births	1753	40.8 (39.4, 42.3)
**Ethnicity**		
Dalit	976	22.7 (21.5, 24.0)
Janjati	1039	24.2 (22.9, 25.5)
Madeshi	36	0.8 (0.6, 1.2)
Muslim	43	1.0 (0.7, 1.4)
Chettri/Brahmin	2065	48.1 (46.6, 49.5)
Other	137	3.2 (2.7, 3.7)
**Mode of birth**		
Vaginal birth (spontaneous, vacuum, forceps)	3694	86.0 (84.9, 87.0)
Caesarean birth	602	86.0 (84.9, 87.0)
**Sex of baby**		
Male	2350	54.7 (53.2, 56.2)
Female	1946	45.3 (43.8, 46.8)
**Birth weight (in grams)**		2920.7 ± 482.8
**Low birthweight**^**a**^		
No ≥2500 g	3778	88.1 (87.1, 89.0)
Yes < 2500 g	510	11.9 (11.0, 12.9)
**Gestational age (in weeks)**		
**Preterm birth**^**a**^		38.6 ± 3.4
No, ≥37 completed weeks gestation	3901	90.9 (90.1, 91.8)
Yes, < 37 completed weeks gestation	387	9.0 (8.2, 9.9)

EN-BIRTH participants (*n* = 4296) were a subset from the NePeriQIP study (*n* = 6929) and demographic characteristics for both are shown in Additional file 3.

Ethnic groups with socio-economic advantages include: Chettri/Brahmin and other; disadvantaged ethnic groups include Dalit, Janjati, Madeshi, Muslim.

^a^Missing values = 8

**Table 2:** Coverage of respectful maternity care during childbirth, EN-BIRTH study (*n* = 4296)

Disrespect and abuse typology		Respectful Maternal and Newborn Care	Coverage (95% CI)
No abuse	1 to 3	Woman or baby not abused (physically, verbally or sexually) during labour or childbirth or after birth (n=4296)	100.0%
No stigma and discrimination	4.1	Woman and baby treated with respect and dignity (n=4296)	100.0%
	4.2	Health service met religious and cultural birthing practice needs (n=3252)	87.0% (85.9–88.0)
	4.3	Mother was satisfied with the privacy during her stay at the hospital (n=3622)	84.3% (81.9–86.7)
Met standards	5.1	Baby kept in skin-to-skin contact with mother immediately after birth (n=803)	18.7% (17.6–19.9)
	5.3	Medical doctor examined the baby in presence of the mother (n=4292)	99.9%
Rapport between women and providers – effective communication	6.1	Woman was satisfied with the health education and information received from health care providers (n=4296)	100.0%
	6.5	Woman were given the opportunity to discuss any concerns and preferences (n=4296)	100.0%
	6.7	Woman received written or verbal information and counselling on exclusive breastfeeding until 6 months before discharge (n=4296)	100.0%
	6.8	Woman received written or verbal information and counselling on nutrition and how to eat healthily (n=13)	0.3% (0.2–0.6)
Health system condition and constraints	7.1	Woman not refused care due to inability to pay (n=0)	0%

### Objective 1: Respectful maternal care

Among the participants enrolled at exit interview (*n* = 4296), there were no reports of any physical, sexual or verbal abuse (Table 2). All women (100%) reported that they had been treated with respect and dignity. More specific questions regarding stigma and discrimination found that 87.0% (95% CI, 85.9–88.0%) of women reported their experience of birth had met religious and cultural needs whilst 84.3% (95% CI, 81.9–86.7%) were satisfied with privacy during their stay in hospital. Satisfaction with health education and information from the health care providers, and the opportunity to discuss any concerns and preferences was 100% at exit-survey report. However, only 0.3% (95% CI, 0.2–0.6%) of women reported receiving written or verbal information/counselling on nutrition or healthy eating. None of the women were refused care because of an inability to pay.

### Objective 2: Respectful newborn care

All women reported that their baby was treated with respect and dignity, with no abuse on exit interview. Reported standards of care were lower with only 18.7% (95% CI, 17.6–19.9%) of women saying that they initiated skin-to-skin contact with their baby immediately after birth (Table 2). 99.9% of women reported that their baby was examined in their presence. All women reported receiving breastfeeding counselling.

### Objective 3: Association between reporting of poor care with socio-demographic and obstetric characteristics

Women identifying as Chettri/Brahmin were most likely to give birth by caesarean section (Additional file 4). Women aged < 20 years (*n* = 563) were most likely to report having their religious and cultural needs met (92.4%, 95% CI 89.9–94.3%) but least likely to report having skin-to-skin contact with their newborns immediately after birth (16.7%, 95% CI 13.8–20.0%), compared to women in other age groups (Table 3). Almost all women who delivered via caesarean section (*n* = 602) reported that their cultural needs had been met (98.3%, 95% CI 96.9–99.1%) and had high satisfaction regarding privacy (97.3%, 95% CI 92.2–100.0%), compared to those with a vaginal birth. Babies born by caesarean were least likely to receive immediate skin-to-skin care (0.5%, 95% CI 0.2–1.5%), compared to those born by vaginal birth (Table 3).

**Table 3:** Coverage of respectful maternity care by socio-economic characteristics, EN-BIRTH study (*n* = 4296)

		Health service met religious and cultural birthing practice needs	Woman was satisfied with privacy during her stay at the hospital	Baby kept in skin-to-skin contact with mother immediately after birth
	n	3252 (95% CI)	3622 (95% CI)	803 (95% CI)
**Woman’s age**				
< 20 yrs	563	92.4 (89.9, 94.3)	88.1 (85.2, 90.5)	16.7 (13.8, 20.0)
20–29 yrs	3149	85.9 (84.6, 87.0)	82.9 (81.6, 84.2)	19.9 (18.6, 21.4)
≥ 30 yrs	584	88.5 (85.7, 90.9)	88.7 (85.9, 91.0)	14.2 (11.6, 17.3)
**Ethnicity**				
Advantaged	2094	88.6 (87.2, 89.9)	86.3 (84.8, 87.8)	17.2 (15.6, 18.9)
Disadvantaged	2202	85.6 (84.1, 87.0)	82.5 (80.9, 84.1)	20.2 (18.6, 21.9)
**Mode of birth**				
Vaginal birth (spontaneous, vacuum, forceps)	3694	85.2 (84.0, 86.3)	82.2 (80.9, 83.4)	21.7 (20.4, 23.1)
Caesarean birth	602	98.3% (96.9–99.1)	97.3% (92.2–100.0)	0.5% (0.2–1.5)
**Parity**				
No previous birth	619	94.5 (92.4, 96.1)	92.4 (90.0, 94.3)	9.9 (7.7, 12.5)
1 previous birth	1924	87.5 (85.9, 88.9)	83.0 (81.2, 84.6)	19.7 (18.0, 21.5)
2 or more previous births	1753	84.0 (82.2, 85.7)	83.1 (81.3, 84.8)	20.8 (19.0, 22.8)
**Sex of baby**				
Male	2350	87.8 (86.4, 89.1)	85.1 (83.6, 86.5)	17.8 (16.3, 19.4)
Female	1946	86.2 (84.6, 87.7)	83.6 (81.8, 85.1)	19.8 (18.1, 21.7)

Ethnic groups with socio-economic advantages include Chettri/Brahmin and other; disadvantaged ethnic groups include Dalit, Janjati, Madeshi, Muslim

Women with no previous births had higher odds of reporting disrespectful care, with an adjusted odds ratio (aOR) of 2.51 (95% CI 1.74–3.61) for reported failures to maintain privacy and 2.20 (95% CI, 1.45, 3.43) for not meeting cultural and religious needs (Table 4). Women who underwent caesarean section were more likely to report privacy was maintained than those who had vaginal birth (aOR 9.44 95% CI 5.41–16.48%). However, babies born via caesarean section had 98% decrease in the odds (aOR = 0.02, 95% CI, 0.01–0.05) of receiving skin-to-skin immediately after birth compared with vaginal births.

**Table 4:** Association between reporting of care with socio-demographic and obstetric characteristics, EN-BIRTH study (*n* = 4296)

	Respectful care				Meeting standards of newborn care	
	Health service met religious and cultural birthing practice needs		Woman was satisfied with privacy during her stay at the hospital		Baby kept in skin-to-skin contact with mother immediately after birth	
	cOR, 95% CI	aOR, 95% CI	cOR, 95% CI	aOR, 95% CI	cOR, 95% CI	aOR, 95% CI
**Woman’s Age**						
< 20 yrs	1.99 (1.44, 2.76)	1.18 (0.80, 1.73)	1.53 (1.16, 2.00)	0.95 (0.68, 1.31)	1.24 (0.98, 1.58)	0.73 (0.54, 0.98)
20–29 yrs	**Reference**	**Reference**	**Reference**	**Reference**	**Reference**	**Reference**
≥ 30 yrs	1.27 (0.97, 1.67)	1.32 (0.99, 1.77)	1.62 (1.23, 2.12)	1.55 (1.16, 2.06)	1.50 (1.17, 1.93)	1.48 (1.14, 1.93)
**Ethnicity (caste)**						
Advantaged	**Reference**	**Reference**	**Reference**	**Reference**	**Reference**	**Reference**
Disadvantaged	1.31 (1.10, 1.57)	1.25 (1.04, 1.50)	1.34 (1.13, 1.58)	1.27 (1.07, 1.50)	1.22 (1.05, 1.43)	1.17 (1.00, 1.37)
**Mode of birth**						
Vaginal birth (spontaneous, vacuum, forceps)	**Reference**		**Reference**		**Reference**	
Caesarean birth	11.43 (5.88, 22.2)	11.27 (5.79, 21.93)	9.82 (5.63, 17.1)	9.44 (5.41, 16.48)	0.02 (0.01, 0.06)	0.02 (0.01, 0.06)
**Parity**						
No previous birth	2.46 (1.70, 3.57)	2.20 (1.45, 3.43)	2.50 (1.82, 3.45)	2.51 (1.74, 3.61)	2.24 (1.68, 2.99)	2.64 (1.89, 3.69)
1 previous birth	**Reference**	**Reference**	**Reference**	**Reference**	**Reference**	**Reference**
2 or more previous births	0.75 (0.63, 0.91)	0.70 (0.57, 0.85)	1.01 (0.85, 1.20)	0.90 (0.74, 1.08)	0.933 (0.79, 1.10)	0.80 (0.67, 0.96)
**Sex of baby**						
Male	0.87 (0.73, 1.04)	0.85 (0.71, 1.02)	0.89 (0.76, 1.05)	0.90 (0.76, 1.06)	0.88 (0.75, 1.02)	0.88 (0.75, 1.03)
Female	**Reference**		**Reference**		**Reference**	

Ethnic groups with socio-economic advantages include Chettri/Brahmin and other; disadvantaged ethnic groups include Dalit, Janjati, Madeshi, Muslim

*cOR*= crude odds ratios, *aOR*= adjusted odds ratios

After adjusting for potential confounders (ethnicity, age, parity and mode of birth), we found that women with no previous births were more likely to report poor care during childbirth (β = − 0.23; *p*-value, < 0.0001), compared with those who had two or more previous births. Women from Madeshi (relatively disadvantaged group) were more likely to report non-respectful care during childbirth (β = − 0.34; p-value, 0.037) than those identifying as Chettri/Brahmin (relatively advantaged group) (Table 5). Women who had caesarean birth had lower reporting of poor care during childbirth (β = − 0.42; *p*-value, < 0.0001) compared with those who had a vaginal birth (Table 5). There was no reported effect regarding the sex of the baby (Tables 4 and 5).

**Table 5:** Predictors for reporting of non-respectful care^a^ during childbirth, EN-BIRTH study (*n* = 4296)

	Uni-variate linear regression				Multi-variate linear regression			
	β	SE	t- value	***p***-value	β	SE	t- value	***p***-value
**Global intercept**	–	–	–	–	0.014	0.08	0.171	0.864
**Woman’s age**								
Intercept	−0.039	0.018	−2.211	0.027	0.33	0.049	6.794	
< 20 yrs	0.173	0.046	3.785	< 0.0001	0.022	0.055	0.395	0.693
20–29 yrs	**Reference**				**Reference**			
≥ 30 yrs	0.123	0.045	2.726	0.006	0.117	0.047	2.507	0.012
**Ethnicity**								
Intercept	−0.031	0.022	−1.412	0.158				
Dalit	0.061	0.039	1.562	0.118	0.051	0.039	1.326	0.185
Janjati	0.112	0.038	2.958	0.003	0.083	0.037	2.213	0.027
Madeshi	−0.369	0.168	−2.2	0.028	−0.344	0.165	−2.082	0.037
Muslim	0.244	0.154	1.591	0.112	0.229	0.152	1.512	0.131
Chettri/Brahmin	**Reference**				**Reference**			
Other	−0.29	0.088	−3.299	0.001	−0.287	0.087	−3.311	0.001
**Mode of birth**								
Intercept	0.366	0.04	9.066	< 0.0001				
Vaginal birth (spontaneous, vacuum, forceps)	**Reference**				**Reference**			
**Caesarean birth**	−0.425	0.043	−9.777	< 0.0001	−0.402	0.044	−9.228	< 0.0001
**Parity**								
Intercept	−0.014	0.023	−0.626	0.532				
**No previous births**	0.242	0.046	5.251	< 0.0001	0.228	0.053	4.321	< 0.0001
1 previous birth	**Reference**				**Reference**			
2 or more previous births	−0.051	0.033	−1.536	0.125	− 0.083	0.035	−2.402	0.016
**Sex**								
Intercept	0.02	0.021	0.993	0.321				
Male	−0.045	0.031	−1.476	0.14	−0.053	0.03	−1.742	0.082
Female	**Reference**				**Reference**			

Ethnic groups with socio-economic advantages include: Chettri/Brahmin and other; disadvantaged ethnic groups include Dalit, Janjati, Madeshi, Muslim

*β*= beta coefficient, *SE*= standard error

^a^Non-respectful care defined as the health service having not met religious and cultural birthing practice needs (*n* = 3252), and that the woman was not satisfied with privacy during her stay at the hospital (*n* = 3622)

## Discussion

In this large-scale study, we attempted to measure the coverage of elements of RMNC during childbirth and look at factors associated with women and newborns not receiving respectful care. The reported prevalence of positive maternity care experiences varied by typology from 0.3–100%. When women were asked about physical, sexual and verbal abuse, none reported the event. Women stated they had been respected during birth in hospital and were satisfied with the information received about their care, their ability to express any concerns, and the health education they received. However, more specific questions around issues that have been widely defined as mistreatment revealed concerns regarding a lack of privacy and religious/cultural needs not being met. No one reported care being denied due to inability to pay, although this is probably because health care for pregnant women and newborns is free at the point of access in Nepal’s public sector.

Given the very high level of satisfaction reported for some questions, we recognise that our findings might reflect the challenges of measuring RMNC in exit-survey. Evidence from Tanzania and Ethiopia suggests that self-reported levels of abuse are lower in facility-based exit interview surveys compared to the levels of disrespect recorded in observation or home-based surveys at a later date [10, 37, 38]. For example, in the same Tanzanian facility, self-reported levels of mistreatment were 9.9%, compared to an observer-assessed prevalence of 69.8%. Instead of reflecting real levels of care, the lower reporting of disrespect in these studies may be related to the proximity of women to the facility and their care givers. Given exit-survey interviews are cheaper and more practical than other forms of research, including home interviews, a better understanding of what can be reliably measured using such tools is needed.

Within our study population, it’s possible that disrespect was “internalized and normalized” by women, and that women did not have high expectations of how they would be treated by health workers [35, 38]. Concepts of respectful maternal – and even more with newborn – care cover a number of components which may, or may not be, considered as ‘disrespectful’ by women. There is an overlap between respectful care, good quality care, and good clinical care that is not always easy to disentangle. In accordance with the ‘*bullseye*’ approach, perceptions of mistreatment can be conceptualised across three main groups: actions garnering wide consensus as disrespectful (e.g. beating a woman), normalized actions constituting mistreatment (e.g. failing to gain informed consent), and structural issues such as deviations from national protocols that women may not even recognize as problematic and might believe represent good quality of care (e.g. application of fundal pressure during the second stage of labour, or being denied food during labour and birth) [39]. Our findings showed respectful care was more likely to be reported by women after caesarean section than those who had a vaginal birth; this could be a manifestation of such structural issues. In Pokhara Hospital, women having caesareans are less likely to share a bed and are monitored more closely in the immediate postnatal period, which may also contribute to an increased feeling of satisfaction with standards of care. In many settings, higher socio-economic status is associated with both a higher prevalence of caesarean section and more respectful care [17]. Measurement tools for RMNC clearly require validation at a local level.

A review mapping evidence around the mistreatment of newborns against seven commonly implemented respectful care typologies exposes critical newborn gaps in these tools and the importance of considering additional categories (such as legal accountability and bereavement care) [19]. Moreover, many research tools assessing respectful care have observations of childbirth stopped shortly after delivery and may therefore exclude critical aspects of respectful newborn care [13, 35, 37, 38]. As aforementioned, evidence from this study suggests key components of what others have defined as respectful newborn care may not be recognized by women as such [40]. Since respectful newborn care is difficult to define and consequently to measure, we suggest agreeing on measurable indicators that make sense to women, such as zero separation, skin-to-skin contact, breastfeeding support, and delayed bathing for 24 h.

Measures of RMNC should also be included as part of service readiness assessments, routine facility-based data for HMIS, and in other health system monitoring and evaluation tools. Measures of birth companionship [41, 42], ability to provide privacy, facility to keep women and newborns together, and availability of a clean environment (including bathrooms) should be considered. There is qualitative evidence from multiple settings that women recognise limitations in health workers’ capacity to provide RMNC, and that not all health facilities provide an enabling environment [43–45]. Lack of infrastructure is an attributing factor to mistreatment [46]. The mistreatment of women is not exclusively caused by incompetent health workers, but is related to systemic health systems and social challenges [47]. Absence of training regarding dignified care, poor infrastructure, high workloads, social and institutional normative values, availability of resources and health system hierarchies can impede provision of respectful care [46, 47]. Responsibility for improving respectful care is not limited to health workers, but is a function of routine health systems, which must be held to account [12]. To this end, measures of service readiness for provision of RMNC should be instituted within standard health facility assessment tools and processes, although currently measures of experience of care are most likely to be excluded [28].

Immediate skin-to-skin contact for newborns is seen as a key component of respectful newborn care [19], but coverage in Nepal was low. Skin-to-skin initiation was lowest for babies born by caesarean (0.5%) compared to those with vaginal/assisted births (21.7%). Delayed initiation of skin-to-skin may be justifiable if general anaesthesia is required and in some clinical emergencies, but for the majority of newborns this represents a critical gap in care [48]. These findings highlight an urgent requirement for improved evidence to support an expanded typology of respectful care that intentionally includes newborns [19], and highlights the importance of disaggregating data by mode of birth. This was a recurrent theme across the EN-BIRTH study [49–52].

There is growing evidence emphasizing the imperative to stratify RMNC data by sociodemographic characteristics, level of education, and ethnicity. In our study, women from advantaged ethnic groups had higher coverage of respectful care than those from disadvantaged groups. A systematic review of 14 studies on disrespect and abuse of women during childbirth in Nigeria showed exposure to abusive behaviours was influenced by low maternal socioeconomic status, lack of education and empowerment of women [15]. In Nepal, like many other settings, caste and ethnicity are key determinants of social hierarchy and access to care [36]. Families from higher castes and relatively advantaged ethnic groups are more likely to receive higher quality of care [34, 53], and have more access to facility birth [54, 55]. Qualitative data to exploring these differences would be helpful to better understand if findings are related to local normative values and potential issues of stigmatisation, or data collection methods.

### Strengths and limitations

This study is an important contribution to the literature assessing measures and measurement approaches to tracking RMNC, especially given the large sample size. All interviews were conducted by female research nurses with standardised training, but there were some limitations. Data were collected using exit-interview survey rather than the gold standard of observation. As discussed, women could have been reporting high levels of respectful care because they were afraid that their answers would get back to the health providers, or because they had such low expectations of care that they were happy with what they received. Respectful care for mothers and newborns is a complex topic and we were not able to explore all facets of the concept within this study, including aspects such as availability of water, food, washroom facilities and latrines. We were not able to measure the socio-demographic characteristics of women, including number of years in education and wealth quintiles, although these have been associated with experiences of disrespect in other settings [10, 35, 42]. While exit-survey interviews are practical and lower in cost, further measurement research using other methods, such as phone or household visit interviews, are needed to gain a better understanding of the reliability of measuring experience of care.

## Conclusions

Reducing mistreatment at birth requires health systems reform to promote and enable respectful care of mothers and newborns around the time of birth. Reliable tracking of valid RMNC measures is imperative to support and accelerate these advances. In our study, as with many others, measuring RMNC by exit interview after hospital birth gave mixed results. All women denied disrespect, abuse and ineffective communication when asked using general questions. Yet more specific detailed questions about stigma and discrimination revealed issues regarding privacy and cultural/religious needs not being met. More research is needed to develop evidence-based measures to track experience of care, including zero separation of mothers and their babies, and to improve monitoring across a range of measurement platforms. Building on these findings, respectful maternal and newborn care should remain a priority in future research.

## Supplementary Information

**Additional file 1.** STROBE checklist.

**Additional file 2.** Questionnaire for respectful maternity care and mapping according to typology, EN-BIRTH study.

**Additional file 3.** Background characteristics of women enrolled in NePeriQIP and EN-BIRTH studies.

**Additional file 4.** Mode of birth by ethnicity at Pokhara Hospital, Nepal, EN-BIRTH study.

**Additional file 5.** Ethical approval of local institutional review boards, EN-BIRTH and NePeriQIP studies.

## Data Availability

The datasets generated during and/or analysed during the current study are available in GC Data repository, http://goldencommunity.org.np/ENBIRTHRMC.

## Abbreviations

aOR: Adjusted odds ratios
CEmONC: Comprehensive emergency obstetric and newborn care
cOR: Crude odds ratios
CIFF: Children’s Investment Fund Foundation
ENAP: *Every Newborn* Action Plan now branded as *Every Newborn*
EN-BIRTH: *Every Newborn*-Birth Indicators Research Tracking in Hospitals study
HMIS: Health management information systems
LMIC: Low- and middle-income country/countries
LSHTM: London School of Hygiene & Tropical Medicine
NePeriQIP: Nepal Perinatal Quality Improvement Project
RMNC: Respectful maternal and newborn care
USAID: United States Agency for International Development
WRA: White Ribbon Alliance
WHO: World Health Organization
