# Supplementary material for: Respectful maternal and newborn care: measurement in one EN-BIRTH study hospital in Nepal
Source: BMC Pregnancy Childbirth. 2021 Mar 26;21(Suppl 1):228. doi: 10.1186/s12884-020-03516-4 (PMC7995692; doi:10.1186/s12884-020-03516-4)
Supplement: Supplementary file 3 — Additional file 3. Background characteristics of women enrolled in NePeriQIP and EN-BIRTH studies. [file 12884_2020_3516_MOESM3_ESM.pdf]

Every Newborn BIRTH multi-country validation study: informing measurement of coverage and quality of maternal and newborn care

Respectful maternal and newborn care: measurement in one EN-BIRTH study hospital in Nepal

Additional File 3: Background characteristics of women enrolled in NePeriQIP and EN-BIRTH studies

		NePeriQIP		EN-BIRTH
	N	Proportion (95% CI)	N	Proportion (95% CI)
**Age (mean ± SD)**	6929	24.6 ± 4.5	4296	24.3 ±4.5
**Woman's age**				
<20 yrs	499	7.8 (7.0, 8.6)	563	13.1 (12.1, 14.1)
20-34 yrs	6226	89.9 (88.6, 90.4)	3617	84.2 (83.1, 85.3)
≥35 yrs	204	2.7 (2.2, 3.2)	116	2.7 (2.3, 3.2)
**Parity**				
No previous birth	954	14.4 (13.4, 15.5)	619	14.4 (13.4, 15.5)
One previous birth	3124	44.8 (43.3, 46.3)	1924	44.8 (43.3, 46.3)
Two or more previous birth	2851	40.7 (39.3, 42.3)	1753	40.8 (39.4, 42.3)
**Ethnicity**				
Dalit, relative disadvantaged	1570	22.7 (21.5, 24.0)	976	22.7 (21.5, 24.0)
Janjati, relative disadvantaged	1743	24.1 (22.9, 25.5)	1039	24.2 (22.9, 25.5)
Madeshi, relative disadvantaged	47	0.8 (0.6, 1.2)	36	0.8 (0.6, 1.2)
Muslim, relative disadvantaged	68	0.98 (0.6, 1.2)	43	1.0 (0.7, 1.4)
Chettri/Brahmin, relative advantaged	3334	48.1 (46.6, 49.6)	2065	48.1 (46.6, 49.5)
Other, relative advantaged	167	3.2 (2.7, 3.8)	137	3.2 (2.7, 3.7)
**Mode of birth**				
Spontaneous vaginal birth	4920	71.0 (69.9, 71.1)	3539	82.4 (81.2, 83.5)
Assisted vaginal birth	214	3.1 (2.7, 3.5)	155	3.6 (3.1, 4.2)
Caesarean birth	1761	25.4 (24.4, 26.5)	602	14.0 (13.0, 15.1)
**Sex of baby**				
Male	3757	54.3 (53.1, 55.5)	2350	54.7 (53.2, 56.2)
Female	3172	45.7 (44.5, 46.9)	1946	45.3 (43.8, 46.8)
**Birth weight (in grams)**	6838	2926.5±507.9		2920.7±482.8
**Low birth weight**				
No ≥2500g	6013	88.2 (87.4, 89.1)	3778	88.1 (87.1, 89.0)
Yes <2500g	804	11.8 (11.1, 12.6)	510	11.9 (11.0, 12.9)
**Gestational age (in weeks)**		39.3±7.2		
**Preterm birth**				38.6±3.4
No ≥37 completed weeks gestation	6342	91.7 (91.0, 92.3)	3901	90.9 (90.1, 91.8)
Yes <37 completed weeks gestation	587	8.3 (7.7, 9.0)	387	9.0 (8.2, 9.9)

References

Day L, Rahman QS, Rahman A, Salim N, KC A, Ruysen H, Tahsina T, Masanja H, Basnet O, Gore-langton G *et al*: Assessment of the validity of the measurement of newborn and maternal health-care coverage in hospitals (EN-BIRTH): an observational study. *Lancet Global* [2020] doi: 10.1016/S2214-109X(20)30504-0.

KC A, Ewald U, Basnet O, Gurung A, Pyakuryal SN, Jha BK, Bergstrom A, Eriksson L, Paudel P, Karki S, et al. Effect of a scaled-up neonatal resuscitation quality improvement package on intrapartum-related mortality in Nepal: a stepped-wedge cluster randomized controlled trial. PLoS Med. 2019;16(9):e1002900.
