# Supplementary material for: Respectful maternal and newborn care: measurement in one EN-BIRTH study hospital in Nepal
Source: BMC Pregnancy Childbirth. 2021 Mar 26;21(Suppl 1):228. doi: 10.1186/s12884-020-03516-4 (PMC7995692; doi:10.1186/s12884-020-03516-4)
Supplement: Supplementary file 4 — Additional file 4. Mode of birth by ethnicity at Pokhara Hospital, Nepal, EN-BIRTH study. [file 12884_2020_3516_MOESM4_ESM.pdf]

Every Newborn BIRTH multi-country validation study: informing measurement of coverage and quality of maternal and newborn care

Respectful maternal and newborn care: measurement in one EN-BIRTH study hospital in Nepal

Additional File 4: Mode of birth by ethnicity at Pokhara Hospital, Nepal, EN-BIRTH study

Ethnicity	Caesarean birth	Instrumental	Vaginal birth
Dalit (n=976)	23.20%	23.20%	23.20%
Janajati (n=1039)	23.10%	27.10%	22.90%
Madhesi (n=36)	0.90%	0.60%	0.90%
Muslim (n=43)	1.00%	0.00%	1.00%
Chhetri/Brahmin (n=2065)	48.30%	46.50%	48.40%
Others (n=137)	3.50%	2.60%	3.50%
