# Supplementary material for: Respectful maternal and newborn care: measurement in one EN-BIRTH study hospital in Nepal
Source: BMC Pregnancy Childbirth. 2021 Mar 26;21(Suppl 1):228. doi: 10.1186/s12884-020-03516-4 (PMC7995692; doi:10.1186/s12884-020-03516-4)
Supplement: Supplementary file 5 — Additional file 5. Ethical approval of local institutional review boards, EN-BIRTH and NePeriQIP studies. [file 12884_2020_3516_MOESM5_ESM.pdf]

Every Newborn BIRTH multi-country validation study: informing measurement of coverage and quality of maternal and newborn care

Respectful maternal and newborn care: measurement in one EN-BIRTH study hospital in Nepal

Additional File 5: Ethical approval of local institutional review boards, EN-BIRTH and NePeriQIP studies

Teams by Country	Institutional Review Boards	Date	Number/Ref
UK	London School of Hygiene & Tropical Medicine (LSHTM) Interventions Research Ethics Committee	03/10/16	11780
Bangladesh	Icddr,b Research review Committee<br>Icddr,b ethical review committee	11/08/16<br>14/11/16	PR 16055
Nepal	Nepal Health Research Council (NHRC)	08/08/16	187/2016
Tanzania	National Institute for Medical Research (NIMRI)<br>Ifakara Health Institute<br>Muhimbili University of Health and Allied Sciences research and Publications committee	20/01/17<br>20/10/16<br>21/10/16	NIMR/HQ/R.8a/Vol IX/2394<br>IHI/IRB/No: 032-2016<br>2016-10-21-/AEC/Vol.XI/310
NePeriQIP study	Nepal Health Research Council	2017	26/2017

Voluntary informed consent was obtained from all participants and their care providers. All women were provided with a description of the study procedures in their preferred language at admission, and offered the right to refuse, or withdraw consent at any time during the study.

This study was granted ethical approval by institutional review boards in all operating counties in addition to the London School of Hygiene & Tropical Medicine.
